# Assessing causal treatment effect estimation when using large observational datasets

**DOI:** 10.1186/s12874-019-0858-x

**Published:** 2019-11-14

**Authors:** E. R. John, K. R. Abrams, C. E. Brightling, N. A. Sheehan

**Affiliations:** 1 Department of Health Sciences, University of Leicester, Leicester, UK; 2 Department of Respiratory Sciences, University of Leicester, Leicester, UK

**Keywords:** Observational data, Causal effect, Instrumental variable, Propensity scores, Unmeasured confounding

## Abstract

**Background:**

Recently, there has been a heightened interest in developing and evaluating different methods for analysing observational data. This has been driven by the increased availability of large data resources such as Electronic Health Record (EHR) data alongside known limitations and changing characteristics of randomised controlled trials (RCTs). A wide range of methods are available for analysing observational data. However, various, sometimes strict, and often unverifiable assumptions must be made in order for the resulting effect estimates to have a causal interpretation. In this paper we will compare some common approaches to estimating treatment effects from observational data in order to highlight the importance of considering, and justifying, the relevant assumptions prior to conducting an observational analysis.

**Methods:**

A simulation study was conducted based upon a small cohort of patients with chronic obstructive pulmonary disease. Two-stage least squares instrumental variables, propensity score, and linear regression models were compared under a range of different scenarios including different strengths of instrumental variable and unmeasured confounding. The effects of violating the assumptions of the instrumental variables analysis were also assessed. Sample sizes of up to 200,000 patients were considered.

**Results:**

Two-stage least squares instrumental variable methods can yield unbiased treatment effect estimates in the presence of unmeasured confounding provided the sample size is sufficiently large. Adjusting for measured covariates in the analysis reduces the variability in the two-stage least squares estimates. In the simulation study, propensity score methods produced very similar results to linear regression for all scenarios. A weak instrument or strong unmeasured confounding led to an increase in uncertainty in the two-stage least squares instrumental variable effect estimates. A violation of the instrumental variable assumptions led to bias in the two-stage least squares effect estimates. Indeed, these were sometimes even more biased than those from a naïve linear regression model.

**Conclusions:**

Instrumental variable methods can perform better than naïve regression and propensity scores. However, the assumptions need to be carefully considered and justified prior to conducting an analysis or performance may be worse than if the problem of unmeasured confounding had been ignored altogether.

## Background

Over the last few years there has been a heightened interest in developing and evaluating different methods for analysing observational data. This has been driven by the increasing availability of large data resources including Electronic Health Record (EHR) data, for example the Clinical Practice Research Datalink (CPRD) in the UK, alongside the recognised limitations of randomised controlled trials (RCTs). Due to the strict eligibility criteria for RCTs their results may not be generalisable to the general population which may lead to a different treatment effect being observed once the treatment is implemented in practice [1]. Additionally, final clinical, and patient-relevant, endpoints can be difficult to obtain in RCTs [2]. These endpoints often require long follow up and large sample sizes, which are not feasible for an RCT due to cost and practical time restrictions. As well as this, RCTs are getting shorter and more streamlined as regulatory bodies, such as the FDA (Food and Drugs Administration) and EMA (European Medicines Agency), wish to accelerate access to innovative health care and technologies [3]. As a result of the increasingly limited evidence that is available from randomised controlled trials (RCTs), NICE (the UK National Institute for Health and Care Excellence) and other policy makers are becoming ever more reliant on observational data to compare the clinical and cost-effectiveness of new treatments to current practice [3]. Due to these issues with RCTs and the improving availability of large EHR data sets, there is an increasing need for researchers to analyse these data appropriately in order to gain additional information about the effectiveness of treatments in clinical practice.

Randomised controlled trials are the `gold standard’ method used to compare the effectiveness of different treatments or exposures since subjects are randomly assigned into different exposure groups rendering the two groups comparable for both known, and unknown, baseline confounders. Because of this comparability, the effect estimates obtained in RCTs can be interpreted as causal effects in that they provide an estimate of the effect of exposure on outcome that is unlikely to be explained by other factors such as confounding or reverse association. Once it is not possible to randomise, the parameter estimates obtained from an observational analysis are associational and may, or may not, have a causal interpretation. Methods have been developed that can disentangle association from causation in an observational setting but these require strong assumptions and can be very sensitive to violations of these assumptions.

The notion of an intervention underlies all approaches to causal inference either explicitly or implicitly. Thus, when we say that an exposure *causes* an outcome, we mean that an intervention on that exposure is informative for the outcome. The problem posed by a causal observational analysis is that of obtaining information on what might happen for a specific intervention when the desired intervention has not taken place [4]. It should be noted that causal methods are not required if the aim is to predict a patient’s risk of disease: in this case association measures would suffice and causal approaches would be inappropriate or potentially misleading. However, when the aim is to intervene, and change a patient’s treatment or exposure, causal approaches are required to understand the `true’ effect of the intervention on the outcome of interest. Our focus is on obtaining reliable estimates of an intervention, by treatment, and so we require causal estimates of the true effect of treatment on outcome.

A wide range of methods are available for analysing observational data. However, various, sometimes strict, and often unverifiable assumptions must be made in order for the resulting effect estimates to have a causal interpretation. These methods need to be evaluated carefully for applications of relevance to health services research in order to assess which assumptions are the most credible in different scenarios. Case studies using real data to compare two, or more, approaches cannot inform whether the resulting estimates are similar because either they are both correct or both incorrect and when the results are different, it is not possible to determine which method is better. For such evaluations, we need to conduct simulation studies where the `true’ effect is known [5]. Appropriate methods for simulating realistic data are hence important to ensure that the nature and distribution of the simulated data are similar to those in the population of interest.

In observational data, patients are not randomised to different treatment or exposure groups and therefore the different groups are often not comparable. Propensity scoring methods are often used to reduce the imbalance between treatment groups using measured baseline covariates [6–8]. The underlying assumption that there are no unmeasured confounders [6] is often not reasonable in observational data.

Instrumental variable (IV) methods can yield causal treatment effect estimates, even in the presence of unmeasured confounding, provided the assumptions of the IV analysis have been satisfied [9–12]. It is known that the level of bias in a two-stage least squares (2SLS) instrumental variables analysis is influenced by the strength of the IV, strength of confounding, and sample size [13–15]. Violations of the assumptions of an IV analysis can also lead to bias in the effect estimates [15, 16]. In previous health services research and health technology assessment studies [13, 15], the simulated data were not based upon patient data. Additionally, only relatively small sample sizes (≤10,000 patients) were considered which were representative of the smaller sample sizes previously observed in clinical research practice [13]. With extensive EHR data now becoming available, and with IV approaches being more widely recommended for the analysis of such data [17, 18], much larger sample sizes are required to assess how such methods would perform in these settings.

The aim of this paper is to revisit some common methods for causal treatment effect estimation in observational data with regard to their performance in big data situations. Our simulations, although simple, are based on an observed cohort of patients with chronic obstructive pulmonary disease (COPD) and assess the appropriateness of 2SLS analysis for different strengths of IV and unmeasured confounding compared with the frequently used approaches of propensity scoring and linear regression. In particular, we wish to quantify the extent to which large sample sizes alleviate some of the recognised problems with IV estimation due to weak instruments, strong unmeasured confounding and small sample bias in a straightforward setting where these methods can, in principle, perform well. More complex settings, such as the analyses of binary and time-to-event outcomes where the IV estimators are often not even theoretically unbiased, will likely pose additional challenges. With the increasing reliance on observational data for treatment effect estimation, it is crucial that researchers understand the underlying assumptions of causal methods and the scenarios for which the different approaches are most appropriate.

## Methods

The target parameter we consider is the average causal effect (ACE) of an exposure *X* on an outcome *Y*. The ACE is a population parameter and is also the target of randomised control trials. The ACE is defined as the difference in expectations for different levels of *X*, where *do*(*X* = *x*) represents an intervention which sets *X* to *x* [19, 20]:

$$ ACE\left({x}_1,{x}_2\right)=E\left[Y|\  do\left(X={x}_1\right)\right]-E\left[Y\ |\  do\left(X={x}_2\right)\right]. $$

If it is assumed that all relationships are linear with no interactions then the dependence of *Y* on *X* and confounders ***C*** can be formulated as in the following equation [16]:

$$ E\left[Y|\  do\left(X=x\right),C=c\right]\kern0.5em =\alpha +\beta \ast X+\gamma \ast \boldsymbol{C}. $$

Under this so called *structural* assumption the ACE is *β*(*x*_1_ − *x*_2_) and so *β* is the causal parameter of interest [11, 16]. The ACE can be estimated using linear regression and propensity scores when all confounders have been measured [6]. Instrumental variable approaches can be used when unobserved confounding is suspected.

### Propensity score

Propensity score methods assume that all confounders have been measured. Let *X* be a treatment variable and ***W*** a set of measured baseline covariates. The propensity score is defined to be the probability of treatment assignment conditional on observed baseline covariates [6, 8]:

$$ e{\left(\boldsymbol{W}\right)}_i=P\left({X}_i=1\ \right|{\boldsymbol{W}}_i\Big). $$

The inverse probability of treatment weighting (IPTW) approach uses weights, obtained from the propensity score, so that the distribution of observed baseline covariates is independent of treatment assignment within the weighted sample [6]. Weighted regression models can then be used to obtain an estimate of the treatment effect. Propensity score stratification, whereby subjects are ranked according to their propensity score and then split into strata based on pre-defined thresholds [6] was also considered.

### Instrumental variables

An IV analysis addresses the case where there are some confounders that are either unknown or unmeasured. For exposure *X* and outcome *Y*, let ***U*** represent the set of unmeasured factors confounding the association between *X* and *Y*. For two variables *A* and *B*, the notation *A* ⊥ *B* denotes that *A* is independent of *B*. For a variable *Z* to be an IV it needs to satisfy the following three conditions:

- *Z* is associated with *X*
- *Z* affects the outcome *Y* only through *X* or, more formally, *Z* ⊥ *Y* ∣ *X*, ***U***
- *Z* is independent of unmeasured confounders ***U***

**Fig. 1:**
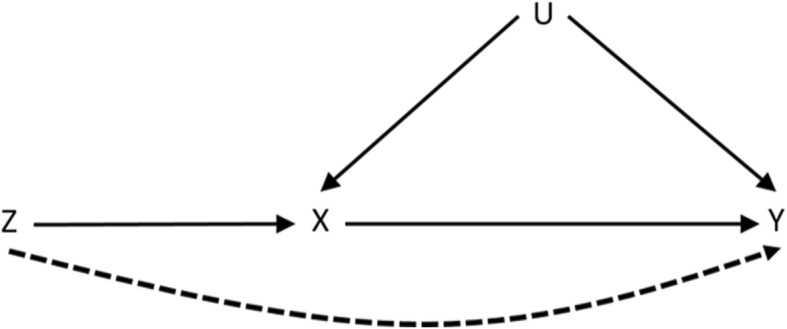
Directed acyclic graph (DAG) representing the conditional (in)dependencies implied by the IV core assumptions. The dashed line represents a violation of condition (b) whereby there is a path from the instrument *Z* to the outcome *Y* that does not go through *X*

These conditional (in)dependencies are uniquely represented in the directed acyclic graph (DAG) in Fig. 1. Note that only the first of these can be verified empirically as the others involve the unmeasured confounding ***U*.**

#### Two-stage least squares

In order to obtain a point estimate for the ACE, additional assumptions must be made. The 2SLS procedure is one of the more popular IV approaches to estimating the ACE [11]. Here, we assume that all relationships outlined in the DAG in Fig. 1 are linear with no interactions [16]. If *Y*_*i*_, *Z*_*i*_ and *X*_*i*_ denote the outcome, IV and exposure for each individual *i* respectively, 2SLS proceeds as follows:

1. Regress *X* on *Z* by least squares to obtain fitted values $\hat{X}$
2. Regress *Y* on $$ \hat{X} $$.

2SLS can be extended to adjust for measured covariates ***W*** in the data.

Under the structural assumption, the above approach targets the average causal effect which is defined in terms of changes across the whole population and is the target of an RCT. Sometimes it is of interest to consider *local* causal effects, especially when there is effect modification whereby individuals in different subgroups, defined by age for example, respond differently to exposure or intervention. Moreover, the classical model (above) is implausible in many situations especially when *Z* and *X* are both discrete [11], although it may be a reasonable approximation. Two particular local parameters are popular and can be targeted under weaker assumptions. The effect of exposure on the exposed (or the effect of treatment received) can be identified under the conditions of an additive structural mean model which, unlike the linear no interactions model, makes no assumptions about the role of ***U*** provided there is no effect modification by *Z*. This parameter is useful in econometrics for evaluating effectiveness of training schemes that involve voluntary participation, for example. The bias induced by self-selection into the scheme means that reliable estimation of the ACE is not possible without additional, potentially untestable, assumptions. Similarly in an RCT with invalid randomisation, such as when seriously ill patients have the right to be given the experimental treatment, estimates of the desired population parameter will be confounded by the patients’ attitudes and/or health while the local parameter can provide some useful information on the effectiveness of the treatment [21]. With valid randomisation, IV core conditions (b) and (c) can be replaced by the *exclusion restriction* stating that *Z* has no causal effect on *Y* other than through *X*. This, together with a monotonicity assumption (that there are no defiers) is sufficient to identify the *complier* causal effect which is the effect of treatment assignment on a population with comparable compliance behaviour. Compliers are those patients who would follow their assigned treatment regardless of which treatment they were assigned to whilst defiers are those patients who will always take the opposite of what they are assigned to. The set of `compliers’ is an unidentifiable subgroup and is IV-dependent. There are also issues with interpreting this parameter when the IV is not causal, as it is implicitly assumed to be in the potential outcomes framework: compliance is then defined with respect to some latent causal factors associated with the IV. For these reasons, it is argued that the complier causal effect is not always an ideal parameter to target for decision-making purposes [4, 9–12, 22–25].

### Simulation study

A simulation study was conducted based upon a small dataset of patients with COPD containing less than 100 patients across the two treatment groups [26, 27]. The outcome of interest was the percentage change in FEV1 (forced expiratory volume in 1 s) between the initial exacerbation visit and the follow-up visit at 2 weeks. The exposure of interest was treatment with steroids and antibiotics versus treatment with steroids alone. Unmeasured confounding of the treatment-outcome association was suspected.

The IV proposed for this analysis was sputum type. Sputum type was classified into two categories: mucoid and mucopurulent. Mucoid sputum is a clear watery substance, mucopurulent sputum is thicker and yellowy in colour. Clinical knowledge indicated that an increase in purulence of sputum is indicative of an infection and should increase the subject’s likelihood of being prescribed antibiotics. Clinical opinion indicated that sputum colour should not affect the outcome, change in FEV1 after 2 weeks, other than via treatment. However, the possibility of a backdoor path through the unmeasured confounders could not be completely ruled out. Any such backdoor path was deemed likely to be very weak compared to the response to intervention. The following baseline characteristics were simulated based on observed values in the real data: body mass index (BMI), time since diagnosis of COPD (TimeCOPD) and previous hospitalisations (Hospitalisation). These three variables were simulated based on features of their joint distribution inferred from the real dataset.

#### Dataset generation

The measured covariates were generated using the observation that the joint distribution for three variables, *A* (BMI), *B* (TimeCOPD) and *C* (Hospitalisation), can be factorised as:

$$ P\left(A,B,C\right)=P\left(A|B,C\right)\ P\left(B|\ C\right)\ P(C). $$

Specifically, continuous BMI was generated from a normal distribution with the mean and standard deviation taken from the actual COPD dataset. The normal distribution was truncated, using the truncnorm package in R, taking values roughly based on the minimum and maximum in the observed data. The binary variable Hospitalisation was generated to be dependent upon the following three BMI categories: healthy, overweight and obese. Hospitalisation was obtained using the proportions in each BMI category taken from the COPD dataset. Continuous TimeCOPD was set to be dependent upon both Hospitalisation and BMI. TimeCOPD was generated separately for each combination of the different BMI and Hospitalisation categories. For each combination, TimeCOPD was taken from a normal distribution with the mean and standard deviation taken from the respective BMI and Hospitalisation distributions. The normal distributions were truncated using values roughly based on the minimum and maximum in the observed data. The continuous variables BMI and TimeCOPD were then centred around their respective means. A normally distributed variable *U*, with zero mean and standard deviation 1, was created to represent unmeasured confounding. The binary instrumental variable, sputum type (*Z*), was simulated using the proportions observed in the COPD dataset. The values taken from the COPD dataset and used in the simulation are given in Table 1.

**Table 1:** Parameter values, taken from a dataset of patients with Chronic Obstructive Pulmonary Disease (COPD), used to simulate the baseline covariates. BMI, Body Mass Index; TimeCOPD, Time since diagnosis of COPD; SD, Standard Deviation

Baseline Covariate	Mean (SD)	Min – Max	Proportion (%)
**BMI**	25.62 (4.61)	16.00–40.00	–
**Hospitalisation (Hosp = 1)**			
Healthy	–	–	40.00
Overweight	–	–	36.00
Obese	–	–	26.70
**TimeCOPD**			
No admission, Healthy	9.59 (9.21)	0.10–35.00	–
No admission, Overweight	9.13 (8.00)	0.10–30.00	–
No admission, Obese	7.25 (9.92)	0.10–25.00	–
Admission(s), Healthy	8.20 (8.03)	0.10–30.00	–
Admission(s), Overweight	3.21 (2.47)	0.10–10.00	–
Admission(s), Obese	5.67 (3.87)	0.10–15.00	–
**Sputum Type (Z = 1)**	–	–	77.65

The exposure, treatment allocation (*X*), and the outcome, percentage change in FEV1 (*Y*), were then generated based on the simulated baseline characteristics and unmeasured confounding. Binary treatment was simulated to be dependent on the IV and unmeasured confounding using probabilities taken from a probit distribution (1). The treatment for each participant *i* was then generated by drawing from a binomial distribution with probability *trtprob*_*i*_ (2):

1 $$ trtpro{b}_i=\Phi\ \left({\alpha}_0+{\alpha}_1\ast {Z}_i+{\alpha}_2\ast {U}_i\right) $$

2 $$ {X}_i\sim Binom\left(1, trtpro{b}_i\right) $$

where *X*_*i*_ = 1 represents the steroid and antibiotic treatment group and *X*_*i*_ = 0 the steroid only treatment group. Note that the 2SLS IV analysis assumes the relationship between treatment and the IV to be linear despite both being binary variables. This may be a poor approximation but 2SLS is quite robust to misspecification of the `first stage’ regression model, especially if measured covariates have been accounted for [28]. In an attempt to induce imbalance between the treatment groups, treatment was also simulated to be dependent upon the covariates BMI, Hospitalisations and TimeCOPD, in addition to sputum type. The results of the propensity score analysis were very similar to those obtained when treatment probability was simulated as above and so are not presented here.

The outcome, percentage change in FEV1 (*Y*) was then generated to be dependent on treatment, the baseline covariates and unmeasured confounding (3).

3 $$ {\displaystyle \begin{array}{c}{Y}_i={\beta}_0+{\beta}_1\ {X}_i+{\beta}_2\ \left({BMI}_i-E\left[ BMI\right]\right)\\ {}+\kern0.5em {\beta}_3\ \left({TimeCOPD}_i-E\left[ TimeCOPD\right]\right)\\ {}+{\beta}_4\ {Hospitalisation}_i+{\beta}_5\ {U}_i+{\varepsilon}_i\end{array}} $$

where *ε*_*i*_ ∼ *Normal*(0, *σ*^2^). Under the assumptions of linearity and no interactions, *β*_1_ is the causal treatment effect parameter we wish to recover [16]. The parameters *β*_0_, *β*_2_, *β*_3_, *β*_4_ and *σ*^2^ in (3) were obtained from a linear regression of the outcome on the baseline covariates and treatment in the real COPD data set. These values are provided in the caption of Table 2. In the COPD study, patients had >50 % chance of being treated with steroids and antibiotics regardless of which sputum class the patient was in. Sputum type was hence a weak predictor of treatment which is often the case for non-randomised IVs. The parameter *α*_0_ in (1) was set at 0.3 to give a baseline probability of treatment similar to that observed in the COPD data. The summary measures of the simulated covariates compared well with those from the real COPD data and so seemed reasonably realistic.

**Table 2:** Parameter values used in different simulation scenarios. The following parameters remained fixed across all scenarios: *α*_0_ = 0.3, *β*_0_ = 17.480, *β*_2_ = 1.335, *β*_3_ = 0.493, *β*_4_ = 14.007, *σ*^2^ = 10.0

Scenario	Parameter Value
**Strength of IV**	
***α***_**1**_	0.1, 0.3, 0.5, 0.8, 1.0
**Strength of Confounding**	
***α***_**2**_	0.0, 0.1, 0.3, 0.5, 0.8
***β***_**5**_	0.0, 1.0, 5.0, 10.0
**Causal Treatment Effect**	
***β***_**1**_	0.5, 1.0, 2.0, 3.0, 5.0
**Direct Effect**	
***β***_**6**_	0.0, 0.1, 0.3, 0.5, 1.0

#### Scenarios to be investigated

A weak IV is an instrument that does not explain much of the variability in the exposure *X* [14]. Different strengths of IV were assessed by varying the *α*_1_ parameter. The strength of unmeasured confounding of the treatment-outcome association on the results of the IV analysis was assessed by varying *α*_2_ and *β*_5_. The strength of causal treatment effect *β*_1_ was varied throughout the simulation study. An additional parameter *β*_6_ was introduced to (3) to assess the effect of a direct path between the IV and outcome (see Fig. 1). In this last scenario the outcome, was generated using (4):

4 $$ {\displaystyle \begin{array}{c}{Y}_i={\beta}_0+{\beta}_1\ {trt}_i+{\beta}_2\ \left({BMI}_i-E\left[ BMI\right]\right)\\ {}+{\beta}_3\ \left({TimeCOPD}_i-E\left[ TimeCOPD\right]\right)\\ {}+{\beta}_4\ {Hospitalisation}_i+{\beta}_5\ {U}_i+{\beta}_6\ {Z}_i+{\varepsilon}_i\end{array}} $$

The parameter values used for the different simulation scenarios are given in Table 2. Combinations of these parameters were also considered to see the effect of varying more than one factor at the same time.

#### Sample and simulation size

Datasets with 2 000, 20 000 and 200 000 patients were created. Even the smallest of these is much larger than the original dataset upon which this simulation study is based. Two hundred simulated data sets were generated for each sample size and scenario under investigation. All simulations were run using the statistical software package R.

#### Analysis models fitted

##### Adjusted linear regression

An adjusted linear model was fitted to give a naïve estimate of the treatment effect. Under the strong and unverifiable assumption of `no unmeasured confounding’, this would be an estimate of the causal effect of treatment. The fitted linear model adjusted for all measured covariates is:

5 $$ {Y}_i={\gamma}_0+{\gamma}_1\ {X}_i+{\gamma}_2\  BM{I}_i+{\gamma}_3\  TimeCOP{D}_i+{\gamma}_4\  Hospitalisatio{n}_i. $$

##### Propensity score

Propensity score models were fitted incorporating the baseline covariates BMI, Hospitalisation and TimeCOPD since they are predictive of the outcome. Sputum type was only predictive of exposure, and not outcome, so was not included as this could lead to amplified bias in the propensity score regression results [29, 30]. The propensity score (*e*_*i*_) was fitted using a logistic regression of the exposure *X* on the baseline covariates:

6 $$ {e}_i=\frac{1}{1+\mathit{\exp}\left({\tau}_1\  BM{I}_i+{\tau}_2\  TimeCOP{D}_i+{\tau}_3\  Hospitalisatio{\mathrm{n}}_i\right)} $$

IPTW propensity score weights (*T*) were calculated using the formula in (7) for exposure *X*_*i*_ and propensity score *e*_*i*_ and incorporated into a weighted linear regression model.

7 $$ {T}_i=\frac{X_i}{e_i}+\frac{1-{X}_i}{1-{e}_i} $$

##### Instrumental variables

Unadjusted 2SLS IV models were fitted with robust standard errors [9] to give an estimate of the average causal treatment effect on the outcome. The first and second stage regression models are given in Eqs. 8 and 9 respectively:

8 $$ E\left[X|\ Z\right]={\alpha}_0\kern0.5em +{\alpha}_1\ Z $$

9 $$ E\left[Y\right]={\beta}_0+{\beta}_1\ \hat{E}\left[X|Z\right] $$

2SLS IV models adjusting for the measured covariates were also fitted.

#### Outcome and summary measures

All models were compared on 200 simulated data sets in each scenario. The following outcome measures were recorded for each model and dataset:

1. Treatment effect estimate: ${\hat{\beta}}_1$
2. Bias: $$ Bias\left({\hat{\beta}}_1\right)={\hat{\beta}}_1-{\beta}_1 $$
3. Z-statistic: $$ {Z}_{stat}=\frac{{\hat{\beta}}_1-{\beta}_1}{SE\left({\hat{\beta}}_1\ \right)} $$
4. Mean squared error: $MSE\left({\hat{\beta}}_1\right)=\kern0.5em E{\left[\Big({\hat{\beta}}_1-{\beta}_1\right)}^2\Big]= Var\left({\hat{\beta}}_1\right)+ Bias{\left({\hat{\beta}}_1,{\beta}_1\right)}^2$

where *β*_1_ is the known `true’ causal treatment effect parameter used in the simulation. *SE*(*β*_1_) is the standard error of the parameter effect estimate from each model.

These outcome measures were summarised across all simulations using the sample mean and Monte-Carlo standard deviations. Coverage, defined as the proportion of the 200 simulated data sets that had a 95% confidence interval containing the true effect estimate *β*_1_, and power to detect a treatment effect, defined as the proportion of the 200 simulated data sets with a 95% confidence interval that did not contain zero, were also reported.

## Results

### Initial parameter values

Initially the data were simulated using a relatively strong IV (*α*_1_ = 0.5), with a small level of unmeasured confounding of the treatment-outcome association (*α*_2_ = 0.3, *β*_5_ = 1.0) and a moderate treatment effect (*β*_1_ = 3.0). The results are presented in Table 3. The adjusted linear regression model was biased, but very precise, at all sample sizes. Coverage was poor with none of the 95% confidence intervals covering the true treatment effect estimate but power was high. The IPTW propensity score approach yielded exactly the same results as the adjusted linear regression model.

**Table 3:** Summary measures for the initial parameter values: *α*_1_ = 0.5, *α*_2_ = 0.3, *β*_5_ = 1.0. The causal treatment effect was *β*_1_ = 3.0. Results are across 200 simulated data sets; values are: sample mean (Monte Carlo SD) unless otherwise stated

	*N* = 2000	*N* = 20,000	*N* = 200,000
**Adjusted Linear Model and Propensity Score IPTW**			
Effect Estimate	3.47 (0.05)	3.47 (0.02)	3.47 (0.01)
Bias	0.47 (0.05)	0.47 (0.02)	0.47 (0.01)
Mean Square Error	0.23 (0.04)	0.22 (0.02)	0.22 (0.00)
Z Statistic	9.41 (0.92)	29.97 (1.05)	94.92 (1.06)
Coverage: n (%)	0 (0.00)	0 (0.00)	0 (0.00)
Power: n (%)	200 (100.00)	200 (100.00)	200 (100.00)
**2SLS IV**			
Effect Estimate	2.62 (2.53)	3.06 (0.73)	2.98 (0.24)
Bias	−0.38 (2.53)	0.06 (0.73)	−0.02 (0.24)
Mean Square Error	12.90 (10.63)	1.06 (0.84)	0.12 (0.07)
Z Statistic	−0.12 (0.90)	0.08 (0.87)	−0.07 (0.93)
Coverage: n (%)	192 (96.00)	194 (97.00)	197 (98.50)
Power: n (%)	27 (13.50)	191 (95.50)	200 (100.00)
**Adjusted 2SLS IV**			
Effect Estimate	3.03 (0.30)	3.00 (0.10)	3.00 (0.03)
Bias	0.03 (0.30)	0.00 (0.10)	0.00 (0.03)
Mean Square Error	0.18 (0.16)	0.02 (0.01)	0.00 (0.00)
Z Statistic	0.15 (0.87)	0.04 (1.00)	−0.07 (1.04)
Coverage: n (%)	194 (97.00)	192 (96.00)	187 (93.50)
Power: n (%)	200 (100.00)	200 (100.00)	200 (100.00)

The unadjusted 2SLS IV model was biased at small sample sizes with fairly high variability (SD ≥ 2.50) across the effect estimates. The uncertainty in the effect estimates led to large bias and very low power to detect a statistically significant treatment effect at small sample sizes (*N* = 2000). The bias and variability in the effect estimate reduces as the sample size increases leading to an increase in both the power and coverage of the effect estimates. Adjusting for measured covariates in the 2SLS IV model led to a large reduction in the variability of the effect estimates across all sample sizes and also makes the method more robust to misspecification of the first stage regression [28]. The bias of the effect estimates was also reduced, especially at small sample sizes, and power and coverage were both high for larger sample sizes (*N* ≥ 20,000).

### Strength of instrumental variable

The adjusted linear regression model and propensity score models do not involve the IV and therefore had the same level of bias as in the baseline scenario for all strengths of IV.

When a weak IV (*α*_1_ = 0.1) was used, there was bias and variability in the unadjusted 2SLS IV model estimates across all sample sizes. This led to very low power of the unadjusted 2SLS model to detect a significant treatment effect. Bias improved with larger sample sizes but there was still considerable variability and a power of only 66% even for 200,000 individuals.

**Fig. 2:**
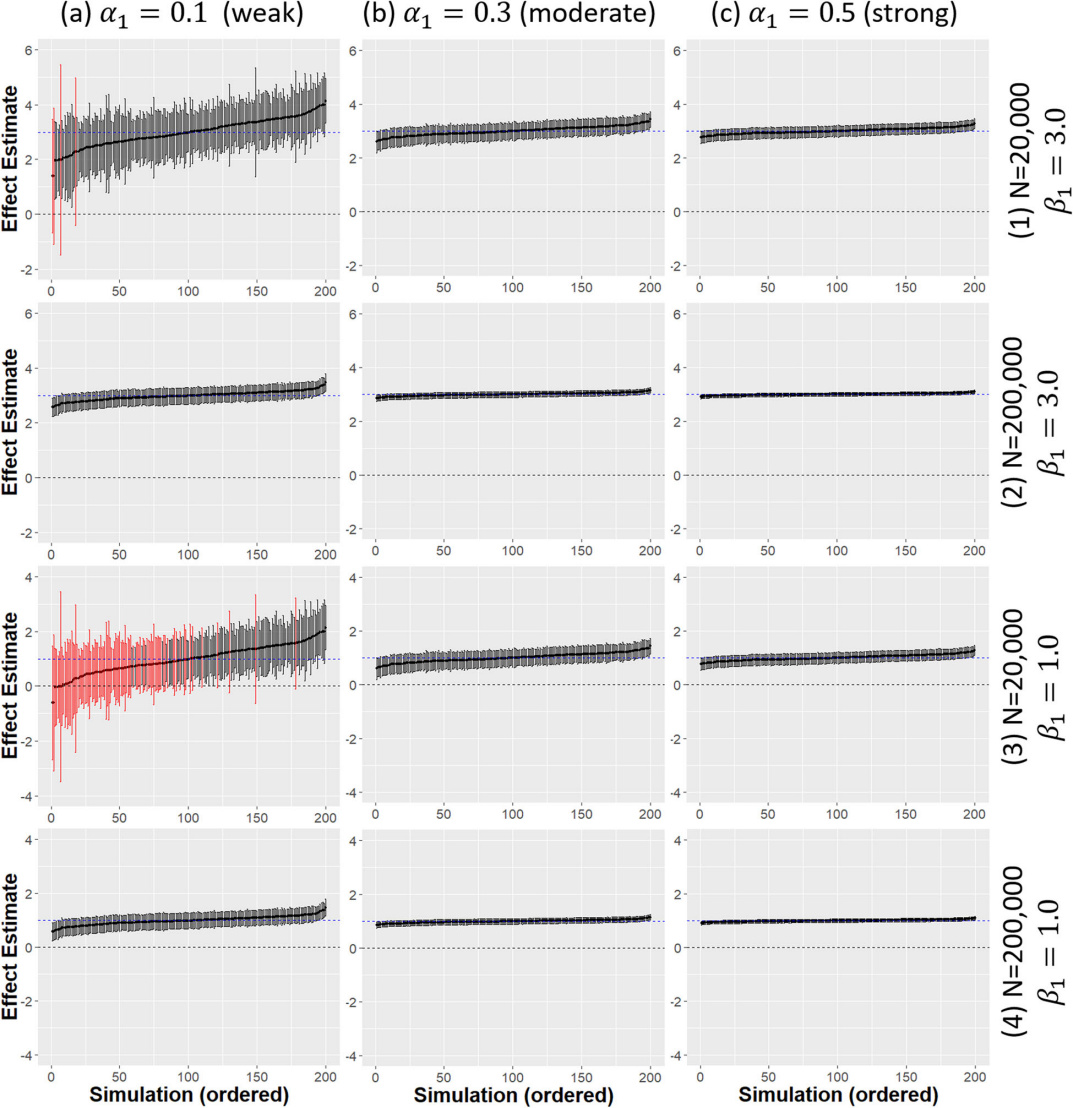
Comparison of adjusted 2SLS IV model estimates from 200 simulated data sets. Different strengths of IV (*α*_1_ = 0.1, 0.3, 0.5) are given across the x-axis (a-c). Different strength of treatment effect (*β*_1_ = 1.0, 3.0) and sample sizes (*N* = 20,000, 200,000) are given across the y-axis (1–4); dashed blue horizontal line is the true causal treatment effect *β*_1_ =3.0, error bars are 95% confidence intervals for the treatment effect estimates for each simulated data set. Black error bars indicate a significant effect estimate, red error bars indicate a non-significant effect estimate

The effect estimates and 95% confidence intervals from the adjusted 2SLS IV analysis are presented in Fig. 2 for different strengths of IV, treatment effect and sample sizes. A weak IV (*α*_1_ = 0.1) led to much greater uncertainty in the effect estimates at all sample sizes compared to when a stronger IV was used even when *N* = 200,000. There was reduced power to detect a significant treatment effect when there was a weak IV (*α*_1_ = 0.1) alongside a weak causal treatment effect (*β*_1_ = 1.0) even for a fairly large sample size (Fig. 2a(3)). In this case much larger sample sizes (*N* = 200,000) were required to obtain a high power (Fig. 2a(4)). Estimate precision was greatly increased for stronger IVs with the causal treatment effect estimates also much closer to the true value. For the smallest sample size considered (*N* = 2,000), both the adjusted and unadjusted 2SLS IV estimates were actually more biased than the linear regression estimates when the IV was weak. 2SLS is known to be affected by finite sample bias and this is exacerbated by a weak IV [14].

The F-statistic, taken from a regression of the exposure *X* on the instrument *Z* can be used as a measure of the strength of an instrument. An F-value greater than 10 is usually taken as an indicator of a `strong’ IV [14, 31]. For the smallest sample size, *N* = 2000 the average F-value was only 2.94 for when *α*_1_ = 0.1 which indicated that this was a weak IV. For larger sample sizes, the *F*-values were greater than 10, however they were still much smaller than in the baseline scenario when *α*_1_ = 0.5.

### Strength of unmeasured confounding

The following combinations of *α*_2_ and *β*_5_ were simulated to give a range of different strengths of unmeasured confounding of the treatment-outcome association:

$$ \left(\genfrac{}{}{0pt}{}{\alpha_2}{\beta_5}\right)=\left(\genfrac{}{}{0pt}{}{0.1}{1.0}\right),\left(\genfrac{}{}{0pt}{}{0.5}{1.0}\right),\left(\genfrac{}{}{0pt}{}{0.8}{1.0}\right),\left(\genfrac{}{}{0pt}{}{0.1}{5.0}\right),\left(\genfrac{}{}{0pt}{}{0.3}{5.0}\right),\left(\genfrac{}{}{0pt}{}{0.5}{5.0}\right),\left(\genfrac{}{}{0pt}{}{0.8}{5.0}\right),\left(\genfrac{}{}{0pt}{}{0.1}{10.0}\right),\left(\genfrac{}{}{0pt}{}{0.3}{10.0}\right),\left(\genfrac{}{}{0pt}{}{0.5}{10.0}\right),\left(\genfrac{}{}{0pt}{}{0.8}{10.0}\right) $$

**Fig. 3:**
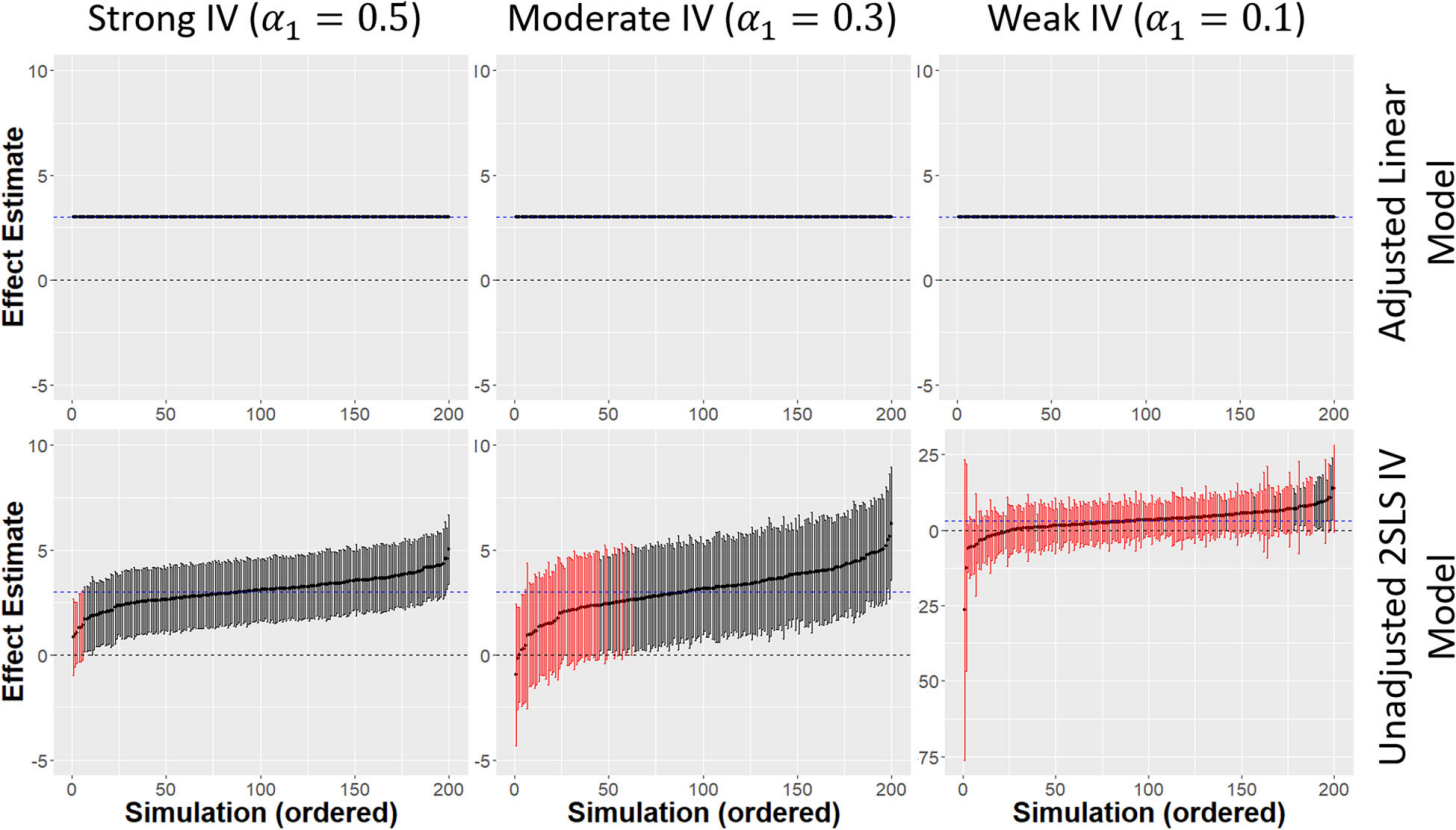
Comparison of adjusted linear model and unadjusted 2SLS IV model estimates from 200 simulated data sets for a sample size of 20,000 and no unmeasured confounding; Different strengths of IV *α*_1_ = 0.1 (weak), *α*_1_ = 0.3 (moderate), *α*_1_ = 0.5 (strong) are given across the x-axis; dashed blue horizontal line is the true causal treatment effect *β*_1_ = 3.0, error bars are 95% confidence intervals for the treatment effect estimates for each simulated data set. Black error bars indicate a significant effect estimate, red error bars indicate a non-significant effect estimate

When there was no confounding (i.e. *α*_2_ and/or *β*_5_ is zero), the linear regression model yielded an unbiased estimate of the causal treatment effect and was less variable than unadjusted 2SLS. This can be seen in Fig. 3 where there is less uncertainty in the linear regression effect estimates compared to the unadjusted 2SLS estimates for all strengths of IV. The uncertainty in the unadjusted 2SLS estimates increased when a weaker IV was used. Adjusting for covariates in the 2SLS regression reduced this uncertainty giving similar results to the linear regression estimates (not shown). As the strength of confounding increased, the bias and variability of the linear regression estimates increased and coverage was poor for all sample sizes, even with weak confounding ($\left(\genfrac{}{}{0pt}{}{\alpha_2}{\beta_5}\right)=\left(\genfrac{}{}{0pt}{}{0.1}{1.0}\right)$). IPTW propensity scoring performed similarly to linear regression for all strengths of unmeasured confounding.

**Fig. 4:**
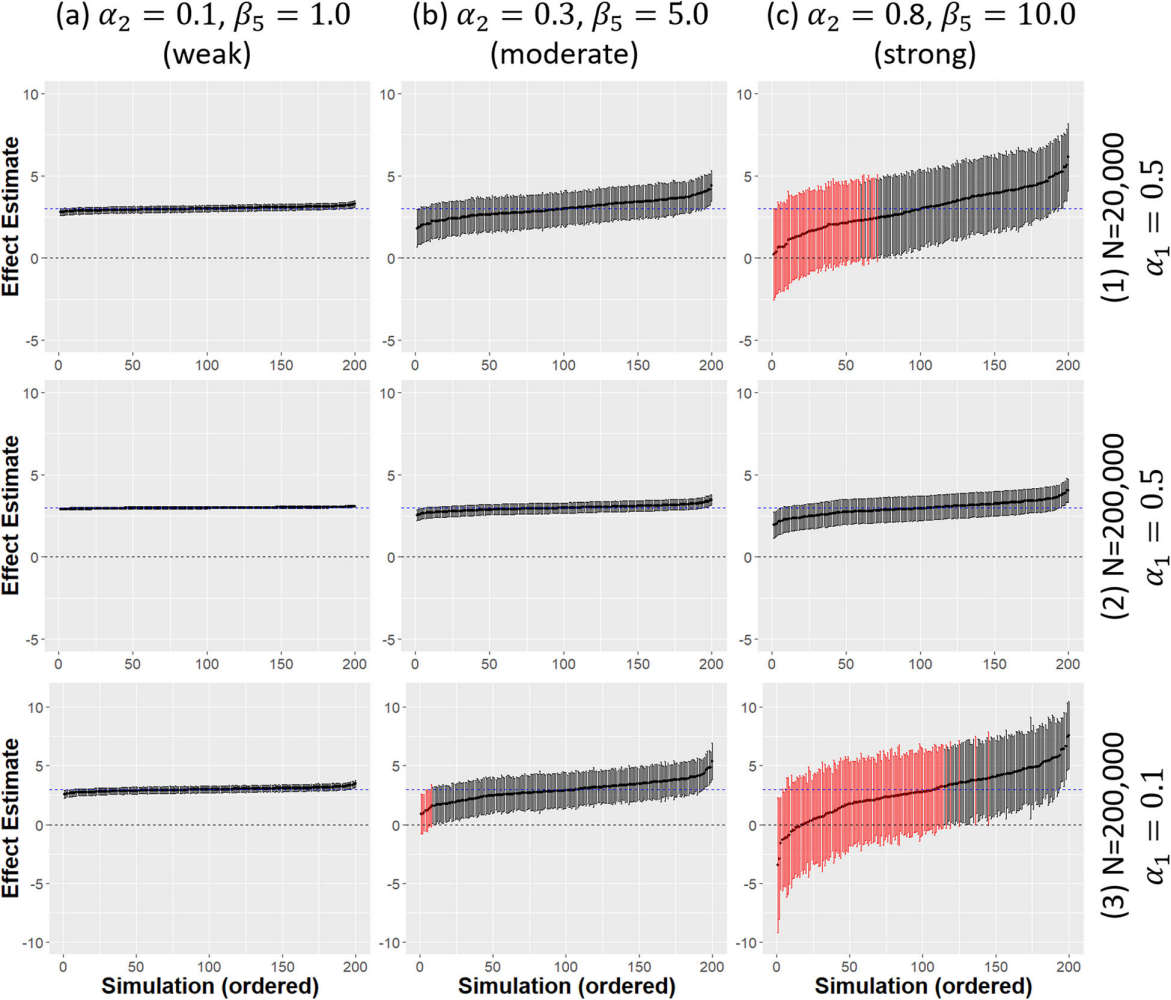
Comparison of adjusted 2SLS IV model estimates from 200 simulated data sets. Different strengths of confounding are given across the x-axis. Two different strengths of IV *α*_1_ = 0.1 (weak) *α*_1_ = 0.5 (strong) and sample sizes (N = 20,000, 200,000) are given across the y-axis; dashed blue horizontal line is the true causal treatment effect *β*_1_ = 3.0, error bars are 95% confidence intervals for the treatment effect estimates for each simulated data set. Black error bars indicate a significant effect estimate, red error bars indicate a non-significant effect estimate

With weak confounding of the treatment-outcome association, and all other parameters at their baseline values, unadjusted 2SLS had power and coverage over 90% once the sample size was reasonably large (*N* ≥ 20,000) and there was minimal bias in the treatment effect estimates. There was much greater variability at the smallest sample size (*N* = 2,000) where unadjusted 2SLS effect estimates were slightly more biased than the linear regression estimates. Adjusting for measured covariates resolved this problem with minimal bias and high coverage and power at all sample sizes (Fig. 4a(1,2)).

Figure 4 shows the effect of increasing the strength of confounding on the adjusted 2SLS effect estimates. Uncertainty in effect estimates increases and power reduces with increasing levels of confounding. Even with a very large sample size, the variability was still greatly increased when there was strong unmeasured confounding although the power remains high.

A weak IV, together with strong confounding, leads to very high uncertainty and large bias in the adjusted 2SLS IV effect estimates. Power to detect a statistically significant treatment effect is low, even at larger sample sizes (*N* = 200,000) as shown in Fig. 4c(3). Performance improves with increasing IV strength but strong confounding causes problems for a moderately strong IV even in large samples. A strong IV (*α*_1_ = 0.8) was required to overcome most of the adverse effects of strong confounding but small sample bias remained an issue.

### Strength of direct effect of the IV on the outcome

Introducing a small direct effect (*β*_6_ = 0.1) of the IV on the outcome to the baseline scenario, and hence violating IV core condition (b), led to biased estimates from both 2SLS IV models at all sample sizes. Adjusting for covariates did not improve performance once there was a direct effect with bias even at large sample sizes (*N* = 200,000). The adjusted linear regression model was also biased at all sample sizes, with a slight increase in bias compared to the baseline scenario, due to the additional unmeasured covariate *Z*. Propensity scoring approaches performed similarly to linear regression in all cases.

Increasing the strength of the direct effect led to an increase in bias in the 2SLS effect estimates across all sample sizes with poorer performance than linear regression once the direct effect size was moderate (*β*_6_ = 0.3). When a stronger direct effect is observed (*β*_6_ ≥ 0.3), there is much more bias in the adjusted 2SLS effect estimates compared to the linear regression model with none of the 95% confidence intervals covering the true effect. Despite the increase in bias, the variability of the adjusted 2SLS effect estimates remained fairly low even when there was a strong direct effect (*β*_6_ ≥ 0.5) leading to precise but inaccurate effect estimates. This can be seen in Table 4 where the standard deviation across the 200 simulations remains less than 0.5 even for a strong direct effect.

**Table 4:** Summary measures for a strong direct effect (*β*_6_ = 0.5). Confounding and strength of IV remained as in the baseline scenario with *α*_1_ = 0.5, *α*_2_ = 0.3, *β*_5_ = 1.0. The causal treatment effect was *β*_1_ = 3.0. Results are across 200 simulated data sets; values are: sample mean (Monte Carlo SD) unless otherwise stated

	*N* = 2000	*N* = 20,000	*N* = 200,000
**Adjusted Linear Model and Propensity score IPTW**			
Effect Estimate	3.55 (0.05)	3.55 (0.02)	3.55 (0.01)
Bias	0.55 (0.05)	0.55 (0.02)	0.55 (0.01)
Mean Square Error	0.30 (0.05)	0.30 (0.02)	0.30 (0.01)
Z Statistic	10.76 (0.94)	34.18 (1.04)	108.28 (1.07)
Coverage: n (%)	0 (0.00)	0 (0.00)	0 (0.00)
Power: n (%)	200 (100.00)	200 (100.00)	200 (100.00)
**2SLS IV**			
Effect Estimate	5.67 (2.52)	6.10 (0.72)	6.01 (0.25)
Bias	2.67 (2.52)	3.10 (0.72)	3.01 (0.25)
Mean Square Error	19.83 (17.89)	10.65 (4.41)	9.17 (1.49)
Z Statistic	0.99 (0.89)	3.69 (0.84)	11.39 (0.90)
Coverage: n (%)	177 (88.50)	8 (4.00)	0 (0.00)
Power: n (%)	118 (59.00)	200 (100.00)	200 (100.00)
**Adjusted 2SLS IV**			
Effect Estimate	6.07 (0.44)	6.04 (0.16)	6.02 (0.05)
Bias	3.07 (0.44)	3.04 (0.16)	3.02 (0.05)
Mean Square Error	9.82 (2.81)	9.32 (0.97)	9.15 (0.30)
Z Statistic	6.03 (0.65)	18.93 (0.73)	60.01 (0.66)
Coverage: n (%)	0 (0.00)	0 (0.00)	0 (0.00)
Power: n (%)	200 (100.00)	200 (100.00)	200 (100.00)

The impact of a direct effect of the IV on the outcome was exacerbated with a weak IV. Here, the 2SLS analyses were a lot more biased than linear regression for all sample sizes considered, even when the direct effect on the outcome was weak. As might be expected, increasing levels of confounding also had a negative effect on performance with increased bias and uncertainty apparent for all sample sizes, and the effect is further compounded when a weak IV was used.

## Discussion

This simulation study verified that, when the instrumental variable and modelling assumptions hold, the 2SLS IV method yielded unbiased estimates in the presence of unmeasured confounding provided that the IV was strong and the sample size was relatively large (*N* ≥ 20,000 in this case). Whilst the precision of the effect estimates increased with increasing sample size, linear regression and propensity score methods remained biased due to the effect of unmeasured confounding. The 2SLS IV method was biased for small sample sizes regardless of the strength of IV or unmeasured confounding. Much larger sample sizes were required when weak instruments were used or when there was strong unmeasured confounding. In particular, strong confounding together with a weak IV could lead to high uncertainty and bias even in very large samples. Whilst adjusting for measured covariates is not theoretically required in order to get an unbiased treatment effect estimate in an IV analysis [9], adjusting always improved performance when the IV was valid [28].

When the assumptions of an IV analysis were violated due to a direct effect of the instrument on the outcome, the 2SLS IV method was biased for all sample sizes. There was also a slight increase in bias of the linear regression and propensity score approaches due to the presence of an additional unmeasured confounder but the 2SLS IV analyses were more sensitive to small increases in the strength of the direct effect. These problems were compounded for weak IVs and strong unmeasured confounding with the 2SLS IV estimates becoming more biased than those from a naïve linear regression which completely ignores the unmeasured confounding.

When there was no unmeasured confounding both linear regression and 2SLS approaches yielded unbiased estimates of the causal treatment effect. However, there was greater uncertainty in the unadjusted 2SLS estimates compared to those from linear regression or propensity score approaches. Therefore, an IV analysis should only be considered when it can be reasonably assumed that the presence of unmeasured confounding is plausible. Otherwise, there is no benefit to using an IV approach over other, simpler, methods such as linear regression that make less stringent assumptions. Of course, modelling assumptions should be checked for all potential analysis methods and the method for which these seem most plausible for a particular application should be employed.

Propensity scoring approaches are commonly used to reduce bias and balance *known* confounding factors between treatment groups in observational data. Whilst a number of different propensity score methods have been proposed, [6, 7, 32, 33], there is some debate as to how well they work in particular situations [34, 35]. They cannot account for unmeasured confounding so they too will yield biased estimates in that case. In our study, propensity scoring methods were found to do no better than a linear regression model. This is perhaps due to our model being truly linear and so the advantages of propensity scores, for non-linear outcomes or in terms of incorporating non-linear terms, were not observed in this setting [6, 34].

Under the assumption of no unmeasured confounders propensity score methods can yield unbiased estimates of the average causal effect. However, if the model for the propensity score is mis-specified this could lead to an inconsistent estimator of the ACE [36]. Alternatively, a regression model for the outcome can be specified based on measured baseline covariates. The ACE is then estimated based on the coefficients from a linear regression which will often be an approximation of the true outcome model. The mis-specification of the outcome model can have a detrimental impact on the bias of the effect estimate if the covariate distributions within the exposed and unexposed treatment groups are very different [37]. Doubly robust estimators have been proposed for causal inference, they are consistent when either the propensity score model for treatment assignment, or the regression model, are correctly specified. These doubly robust estimators give researchers two chances of obtaining an unbiased estimate of the ACE. Simulation studies have shown that doubly robust estimators are more efficient when one of the two models is mis-specified but bias can still arise if both models are incorrect [36, 37]. These estimators should be considered especially when there is high-dimensional confounding. In the simple models considered here, doubly robust methods did not improve on linear regression or propensity score approaches.

When unmeasured confounding is suspected, the 2SLS IV estimator is robust to mis-specification of the first stage regression provided that the second stage is correctly specified [28]. This was observed in our simulations where the first stage regression was assumed to be linear even though the binary treatment values were generated using a probit model. However, the 2SLS IV estimator may not be consistent if the outcome model is mis-specified and the instrument depends non-linearly on the covariates. Locally efficient doubly robust IV estimators have been proposed which are consistent if either the model for the effect of covariates on the outcome, or the model for the instrumental variable given the covariates is correctly specified [38]. Vansteelandt and Didelez [28] have suggested a strategy that will guarantee efficiency of the estimator provided the model for the IV has been correctly specified.

One of the main challenges with instrumental variables analysis is finding an appropriate instrument. It is particularly hard to find a strong IV that is valid (i.e. satisfies assumptions (a)-(c)) when the instrument cannot be randomised by the investigator as is often the case in observational data. There is an upper bound on how strong an IV can be that depends on the strength of unmeasured confounding [31]. Hence, there often is no choice about the strength of IV and researchers cannot be sure that the effect estimates obtained from an analysis with a weak IV are reliable. Furthermore, two of the three IV assumptions ((b) and (c)) cannot be verified empirically from the data as they involve the unmeasured confounder and instead have to be justified from background knowledge which may require consultation and collaboration with relevant experts [12, 16]. In the real COPD data, whilst sputum type appeared to be the most appropriate available IV, the observed association with treatment was unconvincing. This may have been partly due to the very small sample size but it would seem plausible that sputum type is either an invalid, or extremely weak, instrument. While we are willing to believe that sputum type should not affect change in FEV1 after 2 weeks other than via treatment, the possibility of a backdoor path through the unmeasured confounding could not be ruled out. Previous observational analyses have considered physicians prescribing preference, calendar time and genetic variables as instruments but these were not available in the real COPD data [9]. All potential instruments require careful scrutiny with regard to their validity.

Whilst invalid instruments have previously been shown to lead to bias in small sample sizes [15], this analysis shows that larger sample sizes do not alleviate this issue with bias apparent even for the largest sample size (*N* = 200,000) considered. An important message is that an IV approach should not be used if the IV cannot be adequately justified, even if unmeasured confounding is suspected, or the results could be more unreliable than those obtained from a method that ignores the problem and relies on more credible assumptions [11]. IV approaches add an additional layer of assumptions, on top of the relevant modelling assumptions, which are mainly unverifiable from the data. Use of these methods is increasingly being recommended and applied in the medical literature [17, 18, 39] but the analyses are often conducted without checking the relevant assumptions [40]. Moreover, propensity scoring and IV methods are sometimes both employed for the same problem even though they rely on very different assumptions. This can lead to misleading conclusions as discrepancies in the results from the different analysis methods are common [39]. It is therefore crucial that researchers consider the underlying assumptions of all the relevant analysis methods and choose the approach for which these appear to be most plausible.

As is standard in epidemiology, model checking and sensitivity of the conclusions under different model selection and specification should be conducted to assess the robustness of any observed association to various sources of bias [41]. Typically, this requires being able to make an informed judgement about the size of such biases and how to model them. If similar results are observed under several different analysis methods then the conclusions of the study can be viewed as being more robust. When there are discrepancies, understanding the main sources of bias in the different approaches can help to determine what is required in order to answer the causal question. Integrating results from different approaches, relying on different assumptions, is popularly referred to as `triangulation’ [42]. When the IV assumptions cannot be justified, but unmeasured confounding is suspected, sensitivity analysis to the results of non-causal analyses should be conducted. One form of sensitivity, or threshold, analysis considers how strongly an unmeasured confounder would have to be related to both the exposure and the outcome, on the risk ratio scale, in order to explain the observed association without the need for so many assumptions about the unmeasured confounding [43]. An *E-value* can be reported which summarises the minimum strength of association that the unmeasured confounder would need to have with both the exposure and outcome to negate the observational result [43]. The researcher can then consider whether an unobserved confounder of such magnitude is plausible. The smaller the E-value, the less likely it is that the observed association is causal since very little unmeasured confounding would change the result. These approaches can be extended to other scales including continuous outcomes [44, 45]. Sensitivity analyses do not establish existence or absence of a causal effect but they help to clarify how conclusions have been drawn.

This paper focused on a continuous outcome for which instrumental variable methods have been well developed. Issues with non-collapsibility have complicated the generalisation of IV methods to binary and time-to-event outcomes [46, 47]. Further work is required to assess how the issues highlighted above with translate to other outcomes. The problems with bias due to weak IVs, sample size and violations of the assumptions, which arose even in the above simple scenario are likely to be amplified in more complex settings. A perceived limitation of this study is that the simulation only considered a small number of confounding variables. High-dimensional confounding would be more realistic but the relevant effects would also be more complicated and harder to assess. In addition, we did not consider selection bias in this paper. IV analyses are also affected by selection bias. The extent of the bias in IV estimates from non-random samples depends on the selection mechanism. This has been noted in the methodological literature but is not widely acknowledged in practice. Directed acyclic graphs have been recently proposed to depict assumptions about selection and inform sensitivity analyses to determine whether an analysis is biased due to a particular mechanism [48].

## Conclusions

As is evident from our simulation study, the original COPD dataset, with less than 100 patients across both treatment groups, was hugely underpowered to reliably detect a causal treatment effect. Larger sample sizes (such as those derived from EHR data) are becoming more commonplace so issues specifically associated with small samples will not be such a problem in the future. However, a large data set does not necessarily protect from the effects of very weak or invalid IVs even when all the underlying assumptions are satisfied. In particular, it is not always obvious how `large’ it has to be to prevent `small’ sample bias for any particular application. Health services and health technology assessment researchers should think carefully about choice and validation of their instrument before conducting or trusting the results from an IV analysis. In particular, the large sample sizes required for weak IVs have implications for rarer outcomes even in large EHR data sets. In the absence of randomisation, strong assumptions are always required to draw causal, rather than associational, conclusions. Regression and propensity score approaches assume that there is no unmeasured confounding of the treatment-outcome association. IV analyses replace this with equally unverifiable assumptions concerning unmeasured confounding [12]. All methods work well when their assumptions are met. Hence, it is important to consider all analysis methods and adopt the approach for which the assumptions are most plausible for any given application. An IV analysis should never be a default analysis: other methods are better when there is no unmeasured confounding. Furthermore, researchers should consider whether their research question actually requires a causal analysis in the first place, as the results from an inappropriate analysis could be misleading.

## Data Availability

The simulated datasets generated during this study are available from the corresponding author on reasonable request.

## Abbreviations

2SLS: Two-stage least squares
ACE: Average Causal Effect
BMI: Body Mass Index
COPD: Chronic Obstructive Pulmonary Disease
CPRD: Clinical Practice Research Datalink
DAG: Directed Acyclic Graph
EHR: Electronic Health Record
EMA: European Medicines Agency
FDA: Food and Drugs Administration
FEV1: Forced expiratory volume in one second
IPTW: Inverse Probability of Treatment Weighting
IV: Instrumental Variable
NICE: the UK National Institute for Health and Care Excellence
RCT: Randomised Controlled Trial
